# La dysplasie fibreuse ou maladie de Jaffe-Lichtenstein de la clavicule: à propos d'un cas

**Published:** 2012-06-05

**Authors:** Karima Atarraf, Mounir Arroud, Moulay Abderrahmane Afifi

**Affiliations:** 1 Service de traumatologie orthopédie pédiatrique, CHU Hassan II, Fès, Maroc

**Keywords:** Dysplasie fibreuse, clavicule, enfant, Jaffe-Lichtenstein

## Abstract

La dysplasie fibreuse est une maladie osseuse rare, elle représente environ 2,5% des maladies osseuses et 7% des tumeurs osseuses. Nous rapportons le cas d'un enfant âgé de 10 ans, admis pour prise en charge d'une tuméfaction sus claviculaire droite évoluant dans un contexte de conservation de l’état général et chez qui le bilan para clinique a été en faveur de la dysplasie fibreuse de la clavicule.

## Introduction

La dysplasie fibreuse ou maladie de Jaffe-Lichtenstein est une affection sporadique, liée à un défaut de maturation des ostéoblastes à laquelle s'ajoute une activité ostéoclastique anormalement élevée, amenant à une ostéolyse et extension de la maladie. Nous rapportons le 3^ème^ cas mondial de dysplasie fibreuse de la clavicule.

## Patient et observation

**Figure 1:**
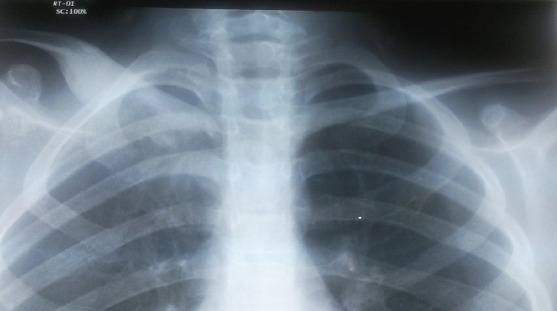
Radiographie standard des clavicules, aspect de condensation osseuse de la clavicule droite avec augmentation de son diamètre, sans lyse osseuse

**Figure 2:**
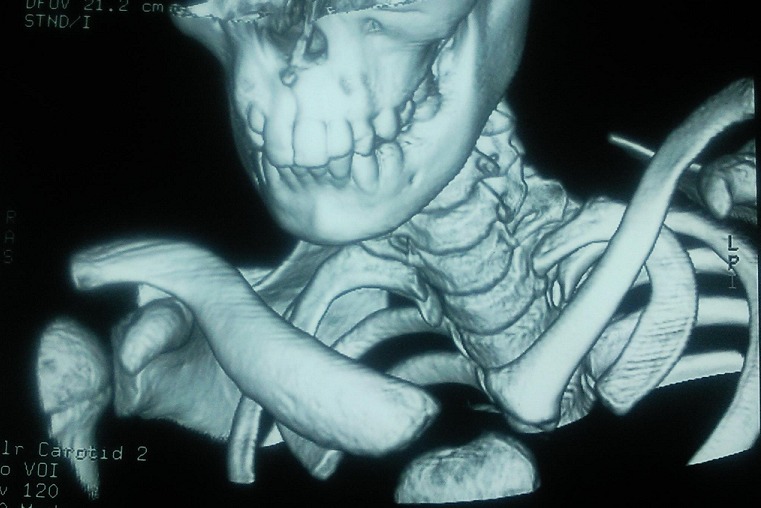
Coupe scanographique objectivant une lésion condensante sans signes de malignité

Enfant H.I., âgé de 10 ans, opéré à l’âge de 2 ans pour hernie inguinale droite. Qui a présente depuis 6 mois une douleur de l’épaule droite avec apparition ultérieure d'une masse sus claviculaire droite de consistance dure, augmentant progressivement de volume sans signes inflammatoires en regard, sans adénopathies axillaires ni sus claviculaires. La radiographie standard des deux clavicules a montré une condensation des 2/3 internes de la clavicule droite, avec augmentation du son diamètre sans lyse osseuse (Figure 1). Le complément scannographique a été en faveur d'une lésion condensante de la clavicule sans signes de malignité (Figure 2). La biopsie osseuse a été réalisée, et l’étude histologique est revenue en faveur d'une dysplasie fibreuse de la clavicule.

Le patient a bénéficié d'un traitement symptomatique fait d'une immobilisation, d'antalgiques et d'anti inflammatoires, Ce qui a contribué à la sédation de la douleur. L’évolution à moyen terme a été marquée par la disparition de la douleur avec diminution de la masse.

## Discussion

La dysplasie fibreuse est caractérisée par les paramètres suivants: Mode de transmission sporadique et en mosaïque; Absence à la naissance; Association au locus chromosomique 20 q13 et au gène GNAS [1]. Elle représente environ 1% des tumeurs osseuses primitives, et 7% des tumeurs osseuses bénignes [2, 3]. Il s'agit d'une pathologie qui touche l'enfant et l'adulte jeune durant la 2ème décade de la vie [4, 5].

Sa forme monostotique est six fois plus fréquente que la forme polyostotique. Elle peut rester Longtemps asymptomatique et souvent de découverte fortuite, souvent révélée par une tuméfaction ou une déformation osseuse [6, 7].

Le sexe féminin représente la moitié des cas de dysplasie fibreuse simple. L'os maxillaire, le fémur proximal et le tibia sont Les os les plus fréquemment touchés, puis de façon moins fréquente l'humérus, le radius et l'os iliaque. L'aspect radiologique conventionnel est celui d'une lésion lytique endomedullaire, diaphysaire ou métaphysaire avec un liseré de sclérose marginale et résorption endostée, le diamètre de l'os est généralement augmenté, et son intérieur est fait d'une matrice ayant la densité d'un verre dépoli. Les lésions peuvent contenir des calcifications arciformes traduisant une calcification progressive des nodules cartilagineux. Ces aspects sont variables sur les os longs et bien plus sur les os plats.

La TDM est cependant utile dans les lésions cranio- faciales permettant de mieux définir l’étendu de la maladie. L'IRM et la scintigraphie osseuse trouvent une indication pour dépister les lésions mal visibles en radiologie conventionnelle.

L’évolution est variable, mais les formes monostotiques dépistées à l'enfance sont en général peu évolutives. La résection chirurgicale du tissu fibreux n'est indiquée que si la maladie est symptomatique, elle reste réservée aux formes volumineuses fragilisant l'os afin de prévenir les fractures pathologique et pour arrêter l’évolution des déformations.

La surveillance des patients doit être prolongée pour dépister au plutôt une dégénérescence sarcomateuse de ces lésions. L'intérêt des biphosphonates en tant que traitement médical reste à prouver.

## Conclusion

La localisation claviculaire de la dysplasie fibreuse monostotique est exceptionnelle. L'imagerie peut être trompeuse, le diagnostic positif est souvent posé grâce à l'histologie.
